# The relationship between fear of Covid-19 and obsessive–compulsive disorder

**DOI:** 10.1186/s40359-023-01112-7

**Published:** 2023-04-26

**Authors:** Maryam Dehghani, Hamideh Hakimi, Maryam Talebi, Hanie Rezaee, Noushin Mousazadeh, Hassan Ahmadinia, Saiedeh Almasi

**Affiliations:** 1 Instructor of Pediatric Nursing Nahavand School of Allied Medical Sciences, Hamadan University of Medical Sciences, Hamadan, Iran; 2 Social Determinants of Health Research Center, Faculty of Nursing and Midwifery, Qazvin University of Medical Science, Qazvin, Iran; 3 Operating Room Technology, Hamadan University of Medical Sciences, Hamadan, Iran; 4 Psychiatry and Behavioral Sciences Research Center, Addiction Institute, Department of Nursing, Amol Faculty of Nursing and Midwifery, Mazandaran University of Medical Science, Sari, Iran; 5 Epidemiology and Biostatistics, Medical School, Occupational Environment Research Center, Rafsanjan University of Medical Sciences, Rafsanjan, Iran; 6 Instructor of nursing, Department of Nursing, Boroujerd School of Nursing, Lorestan University of Medical Sciences, Khorramabad, Iran

**Keywords:** Coronavirus, Fear, Obsessive–compulsive disorder

## Abstract

**Background:**

The coronavirus pandemic and health measures related to it have led to an increase in mental health problems. The relatively high incidence of the disease and its mortality rate created anxiety in society. This study aimed to determine the prevalence of fear of the coronavirus (COVID-19) and its relationship with obsessive–compulsive disorder in patients who attended the outpatient clinic of Besat Hospital in Hamadan.

**Methods:**

In this cross-sectional descriptive study, 320 patients who attended the outpatient clinic of Besat Hospital in Hamadan were selected by random sampling method in 2021. Data were collected using the Fear of the coronavirus (COVID-19) questionnaire and obsessive–compulsive disorder scale and analyzed using SPSS software (V16). They were analyzed using Pearson correlation coefficient and independent t-test.

**Results:**

The mean ± SD age of the subjects was 34.14 ± 9.30 years and 65% of the study subjects were women. The mean ± SD score on the obsessive–compulsive disorder scale was 32.90 ± 19.87 and the mean ± SD score for fear of coronavirus was 16.82 ± 5.79. The contamination dimension of OCD had the highest score of 9.04 ± 5.46 and stealing had the lowest score of 0.10 ± 0.49. The mean fear of COVID-19 in people who had a history of obsessive–compulsive disorder before the quarantine was significantly higher than in those who did not have it (*P* = 0.002). Along with the increasing fear of coronavirus scale score, the score of obsessive–compulsive disorders increased except for the stealing dimension (*P* < 0.001).

**Conclusions:**

The results of the study showed that there was a moderate level of fear of COVID-19 among the study population. Also, a relatively high proportion of study subjects had a weak manifestation of OCD. It seems that two years after the beginning of the Covid-19 coronavirus pandemic, people have adapted to the conditions, and their fear of the disease is reduced.

## Background

In December 2019, cases of acute and severe respiratory infection were reported in Wuhan, China. The disease was the result of a new virus called coronavirus (Covid-19) [1]. The virus spread rapidly around the world. The World Health Organization declared the disease a public health emergency on January 30, 2020 [2, 3]. Iran was one of the first countries in the Middle East which were affected by the virus [4]. We were witnessing a growing trend of the virus in the country [5].

The virus transmission is through respiratory tract secretions, contact with contaminated surfaces, and person-to-person contact [6]. People have tried to protect themselves by taking measures such as social distancing, wearing a mask, and adhering to personal hygiene methods recommended by the World Health Organization [7, 8]. The unpredictability of the situation and the uncertainty about the ending time of the pandemic, the lack of definitive treatment for the disease, and its high contagiosity along with the high mortality rate were the most important reasons for causing anxiety among people. Therefore, this disease, in addition to physical health affects people's mental health at the individual, interpersonal and social levels [9]. A major feature of infectious diseases is the creation of fear in communities. Fear is directly related to the rate of transmission and mortality rate of the disease. Due to the relatively high incidence and mortality of the COVID-19 virus, it caused a lot of fear in people [10].

Factors such as new variants, unknowingness, lack of definitive treatment for the disease, death of loved ones, and recommendations such as frequent hand washing along with the spread of false news and rumors, lead to increased fear of infecting self and others [7, 11, 12]. A fear that is not appropriate to the situation may cause upsetting thoughts and behaviors which can lead to psychological disorders such as panic, depression, anxiety, and obsessive–compulsive disorder [13–16]. Fear of becoming infected with the COVID-19 virus after watching or listening to the news can lead to obsessive behaviors such as excessive hand washing. Being infected with COVID-19 can lead to serious challenges and problems [17]. Previous studies have shown that following instructions such as frequent hand washing (use of antibacterial gel), continuous use of gloves and masks, avoiding physical contact with surfaces and other people (social distancing) to prevent and reduce the risk of COVID-19 infection may increase the risk of development of the obsessive–compulsive disorder [18–20].

Obsessive–Compulsive Disorder (OCD) is one of the most common, debilitating, and resistant to the treatment of psychological disorders. Obsession means thoughts, mental images, and unwanted and frequent desires that are annoying and uncontrollable for the person. Obsessive thoughts often lead to hurting self or important persons. Compulsion is a repetitive physical or mental act that a person forces himself to do to reduce the anxiety caused by obsessive thoughts or to prevent the occurrence of negative consequences [21]. These obsessive thoughts and actions are distressing and time-consuming and interfere with the daily life of the person [6].

In a study, conducted in Italy, during the COVID-19 pandemic, more than one-third of OCD patients reported that their previous obsessions had been intensified. New obsessions also have been developed in these individuals, which led to the worsening of their condition [22]. The results of studies that have investigated the effect of the COVID-19 pandemic on people with OCD reported an exacerbation in their symptoms [23, 24]. People without OCD may experience some mental and behavioral distress during the COVID-19 pandemic. Because over time, most of these behaviors decrease, they should not be considered harmful. However, health providers should be aware of these changes and assess the people who may reach the diagnosis of OCD [25].

The number of people with OCD and fear of COVID-19 infection may increase in the future [19]. After the end of this pandemic COVID-19 pandemic, we are still dealing with its psychological consequences which were caused by fear such as depression and anxiety, and OCD [26]. The experiences of the study researchers who are clinical nurses showed that many patients and their families had obsessive behaviors and thoughts related to fear of developing COVID-19. Nurses, as people who are in direct contact with the patient and members of the community, are responsible for psychological care in addition to physical care. Therefore, in addition to emphasizing the observance of health protocols and social distancing, it is important to pay attention to the impact of fear of coronavirus on the mental health of people in the community. The present study aimed to determine the relationship between fear of Coivd-19 and obsessive–compulsive disorder in patients who attended the outpatient clinic of Besat Hospital in Hamadan.

## Methods

This descriptive cross-sectional study was conducted in 2021. The study sample was 320 patients over 18 years of age who had attended the clinic of Besat Hospital in Hamadan Due to the pandemic, sampling was conducted at Besat Clinic (a General clinic) where people have been attending for minor health problems. Samples were selected using the random sampling method. At first, the researcher referred to the clinic admission department, then samples were chosen from the waiting list randomly. The objectives of the research were explained to the research subjects. Then obtaining informed written consent, and the questionnaires were provided to them. The time to complete the questionnaires was 15 to 20 min.

The first part of questionnaire was related to sociodemographic questions including Age, Gender, Education level, Marital status, using social media, and being diagnosed with OCD. The Fear of COVID-19 Scale which is a self-reporting questionnaire developed by Ahorsu et al. (2020) was used. It includes 7 one-dimensional questions with 5 Likert options (1—Strongly disagree, 2—Disagree, 3—Undecided, 4—Agree, 5—Strongly agree). The scale scores range from 7 to 35. The higher the score, the greater the fear of coronavirus. Internal consistency of this instrument was calculated using Cronbach's alpha (0.82), which indicates the validity of the questionnaire in assessing the fear of Covid-19 in the study population [10]. The Padua Inventory (PI), a self-report measure of obsessive and compulsive symptoms, developed by Leonard Burns et al. (1996) (Washington State University correction) was used to measure obsessive–compulsive disorder. The structural reliability of the scale was confirmed in a study by Shams et al. (2010). They used factor analysis by Principal Axis with varimax rotation. The reliability was confirmed by calculating internal consistency (α = 0.92), Split half with spearman correlation (rho = 0.95) and test–retest correlation (rho = 0.77) [27]. In contrast with other self-report inventories, this questionnaire particularly measures obsessional thoughts/ impulses to harm self/ others, which is not consistent with other OCD questionnaires. The PPI-WSUR is a reliable instrument to measure individual differences in the degree of obsessive–compulsive symptoms in Iran. The validity and reliability of the PPI-WSUR were quite satisfactory. It consists of 39 options that are graded based on the severity of discomfort caused by thoughts or behaviors into 5 scales (no = 0, somewhat = 1, relatively high = 2, high = 3, very high = 4). Dimensions of the Persian Version of the questionnaire include 1. contamination obsessions (6 items); 2. washing compulsions (4 items); 3. ordering compulsions (3 items); 4. checking compulsions (10 items); 5. obsessional thoughts to harm self/others (5 items); 6. obsessional thoughts about violence (2 items); 7. obsessional impulses to harm self/others (7 items); and 8. obsessional impulses to steal (2 items). Scores range from zero to 156 [27].

The collected data were analyzed using descriptive statistics (frequency, percentage, mean and standard deviation). The normality of continuous variables was assessed using the Kolmogorov–Smirnov test and skewness and kurtosis. It had a normal distribution. The results of the Kolmogorov–Smirnov test showed that the distribution of variables was normal. Also, with normal values of skewness and kurtosis (+ 1 to − 1) and a sample size of 320, parametric tests were used. T-test and Pearson's correlation statistical test have been used. The effect of different variables on obsessive compulsive disorder was investigated using multiple linear regression model. The data was analysis by SPSS software version 16.

## Results

In this study, the relationship between fear of Covid-19 and obsessive–compulsive disorder in patients who attended the outpatient clinic of Besat Hospital in Hamadan in 2021 was investigated. The results showed that the mean and standard deviation of age in the research subjects was 14.34 ± 3.9. Some sociodemographic characteristics of the study group are presented in Table 1. 87.2% of subjects used social networks such as news, newspapers, and virtual networks during the quarantine. 9% of the subjects were associated with people with Covid-19 infection. The mean ± SD fear of coronavirus was 5.79 ± 16.82. The prevalence of fear of COVID was 25 percent. Eighty people (25%) of study subjects had scores 21 and above in fear questionnaire which was considered with high level of fear.

**Table 1:** Socio-demographic characteristics

Characteristics	N	%
*Gender*		
Male	112	35
Female	208	65
*Educational attainment*		
Undergraduate	38	11.9
Diploma	76	23.8
Bachelor	155	48.4
higher education	51	15.9
*Marital status*		
Single	124	38.8
Married	196	61.3
*OCD history*		
Yes	28	8.8
No	292	91.3

The mean total score of obsessive–compulsive disorder in research subjects was 19.87 ± 32.90 and its dimensions were lower than the average in all areas. The mean score of contamination obsessions, which is one of the dimensions of obsessive–compulsive disorder, was 9.04 ± 5.46, which was the highest mean among the dimensions of obsessive–compulsive disorder (Table 2). Also, obsessional impulses to steal with a mean of 0.1 had the lowest mean among obsessive–compulsive disorder dimensions. Pearson correlation test showed that the correlation between fear of COVID-19 score and all obsessive–compulsive dimensions was positive and significant (r = 0.53, *P* < 0.001) except for obsessional impulses to steal. With the increase in the fear of COVID-19 score, the score of obsessive dimensions also increased. The effect of the variable fear of corona on obsessive–compulsive disorder is significant (*P* < 0.001). So that if other variables are constant, the increase of each unit of fear of corona has increased the score of obsessive–compulsive disorder by 1.776. Other variables were not predictors of obsessive–compulsive disorder (Table 3).

**Table 2:** Mean and standard deviation of obsessive–compulsive disorder scores in study subjects

Dimensions	Range	Mean ± SD
Contamination obsessions	0–24	5.46 ± 9.04
Washing compulsions	0–16	4.24 ± 6.75
Ordering compulsions	0–12	2.71 ± 3.02
Checking compulsions	0–40	7.76 ± 8.59
Obsessional thoughts to harm self/others	0–20	3.58 ± 2.99
Obsessional thoughts about violence	0–8	147 ± 1.23
Obsessional impulses to harm self/others	0–28	2.59 ± 1.16
Obsessional impulses to steal	0–8	0.49 ± 0.10
Total Score	0–156	19.87 ± 32.90

**Table 3:** The effect of different variables on obsessive–compulsive disorder

Variables	B	SE	Beta	t	Sig
Constant	8.738	5.867		1.489	0.137
Fear of COVID-19	1.776	0.173	0.518	10.257	0.000
Gender (female to male)	− 2.393	2.056	− 0.058	− 1.164	0.245
Educational attainment	− 2.174	1.186	− 0.096	− 1.833	0.068
Marital status (single to married)	1.923	2.043	0.047	0.941	0.347
OCD history	4.531	3.465	0.065	1.308	0.192
Using social networks during quarantine	0.329	2.985	0.006	0.110	0.912
Contact with a person with COVID-19	1.074	1.986	0.027	0.541	0.589

Independent t-test showed the mean fear of COVID-19 was significantly higher in women than men (*P* < 0.002). The scores of washing compulsions in women were higher than in men (*P* = 0.030) and the mean score of obsessional impulses to harm self/others was higher in men than in women (*P* = 0.004). The mean score of obsessional impulses to harm self/others in single subjects was higher than in married subjects (*P* = 0.010).

The mean score of fear of COVID-19 in married subjects was higher than in singles, but the difference between the two groups was not statistically significant (*P* = 0.360). The mean score of fear of COVID-19 in subjects with a history of pre-quarantine obsessive–compulsive disorder was significantly higher than those who did not (*P* = 0.002). The mean scores of the dimensions of obsessive–compulsive disorder such as checking compulsions, ordering compulsions, and obsessional impulses to harm self/others in subjects with a history of pre-quarantine obsessive–compulsive disorder were significantly higher than in subjects without previous history (*P* < 0.05).

The mean score of fear of COVID-19 was higher in those who used social networks during quarantine than in those who did not, but the difference between the two groups was not statistically significant (*P* = 0.228). Also, the mean scores of Obsessional thoughts to harm self/others (*P* = 0.045) and Obsessional impulses to harm self/others (*P* = 0.034) in people who did not use social networks during quarantine, were significantly higher than the group who used.

The mean score of fear of COVID-19 in people who had no contact with a person with COVID-19 was significantly higher than the people who had contact with infected patients (*P* < 0.001). Also, the mean scores of most dimensions of obsessive–compulsive disorder in people who had no contact with a person with COVID-19 disease were higher than the group who had, but this difference was not statistically significant (*P* > 0.05).

## Discussion

In this study, the relationship between fear of COVID-19 and obsessive–compulsive disorder was investigated. The results showed that the level of fear of COVID-19 was moderate among the study population. Almost two years after the COVID_19 pandemic, people seem to have adapted to the situation and the fear of the disease has decreased.

This study is the first one to investigate the relationship between fear of COVID-19 and the occurrence of obsessive–compulsive disorder in the Iranian adult population. In this regard, Seçer et al. conducted a study on the adolescent population of Turkey, which showed that fear of Covid 19 has a positive effect on the development of obsessive–compulsive disorder in adolescents [7]. Other studies have examined the effect of fear of Covid 19 on the severity of symptoms of obsessive–compulsive disorder [28–32]. The results of studies indicate that the symptoms of obsessive–compulsive disorder in patients who had it before have intensified in the COVID-19 pandemic. Thus, the COVID-19 pandemic was a cause of fear and stress for most patients with OCD [29–31]. In previous epidemics such as SARS and MERS, the symptoms of obsessive–compulsive disorder had been intensified in the community [18]. A study in the United States found that during one year of the Covid-19 pandemic, new cases of obsessive–compulsive disorder were increased and the symptoms of the obsessive–compulsive disorder increased in people with a history of the disease [30]. Zheng et al. reported that at the time of the Covid-19 pandemic, people with a history of psychiatric disorders or a positive family history of psychiatric disorders were at higher risk for OCD [32]. The results of a study in India showed that the fear and anxiety caused by COVID-19 did not affect the severity of the symptoms of obsessive–compulsive disorder [28]. Long-term fear of illness causes mental health problems such as stress and anxiety [33]. Stress from covid-19 infection or death can lead to obsessive–compulsive disorder, which in turn forces people to adhere to the protocols obsessively and over-wash things to relieve excessive anxiety.

One study showed that the fear of COVID-19 was higher in densely populated and urban areas of the United States. Also, the level of fear was higher among women and married people with children. People who were more afraid of the COVID-19had more symptoms of anxiety and depression [26]. The present study showed that the fear of COVID-19is more severe in women than men. Other studies have reported higher rates of fear of COVID-19in women than men [34, 35]. This finding is consistent with other reports that indicate that women are more psychologically vulnerable than men during the COVID-19 pandemic. For example, a study by Wang et al. (2020) in 194 Chinese cities found that women were more likely to suffer from stress, anxiety, and depression than men in the COVID-19 pandemic [36]. Women are more affected by the pandemic in terms of personality and emotional characteristics [37]. Because they are more emotional and they have more responsibilities related to marital, maternal, and family care, they experience more stressful life events and a greater fear of COVID-19.

The results of this study showed that marital status did not affect COVID-19fear. In this regard, a study conducted on the fear of COVID-19in the Indian population showed that the average fear of COVID-19in single and married people was not different [38]. While the study by Nino et al. showed that married people were more afraid of COVID-19 than single people [35]. Zheng et al. also reported that single people were more exposed to OCD than married couples during the Covid-19 pandemic [32].

Using information from virtual networks is like a double-edged sword. On the one hand, the use of virtual networks can play a protective role in the deprivation of social relationships. Individuals can use it to maintain social connections and experience fewer mental health problems [39]. While it is good for people to know about the dangers of the virus, on the other hand, exposure to the massive amount of information on virtual networks, which is sometimes inaccurate, unreliable, and even exaggerated, causes fear, stress, anxiety, and depression in healthy people or ones with the previous psychiatric illnesses [40, 41]. The results of some studies have shown that people who use more virtual networks are more susceptible to fear of Covid 19 and severe insomnia [33, 42].

Ahmad et al. also reported that virtual networks increased the fear of Covid 19 and the occurrence of panic attacks [43]. In this regard, the present study also showed obsessional thoughts to harm self/others and obsessional impulses to harm self/others in people who have used virtual networks excessively during the quarantine were higher in comparison to those who use fewer virtual networks.

Although the results of the current investigation showed that there is a significant relationship between OCD and COVID-19 diseases, there are two limitations of the current investigation. First, sampling was performed in the clinic, due to easy access to the community. Being indoors during the pandemic can increase the fear of contracting Covid19 disease. Therefore, it may affect how people answer. The second limitation is related to the sample size, as it is small. Since this study was carried out during the lockdown, the authors were not able to reach a larger number of people. In line with this idea, future work on this topic should include providing online scales. In this case, more people can participate in the study. in addition, it can reduce fear of COVID 19.

The results of this study showed that the fear of coronavirus is higher in married people than in single people, which can affect the psychological dimensions of children. It is suggested that a study be conducted in this field.

## Conclusions

The results of the study indicate that two years after the beginning of the Covid-19 pandemic, the fear of COVID-19is moderate. Many people have mild symptoms of obsessive–compulsive disorder. There is a significant relationship between obsessive–compulsive disorder and COVID-19disease. Although it seems that the People adapted to the situation and the fear of the disease has decreased, its psychological consequences are observed in society. The high percentage of symptoms of obsessive–compulsive disorder is a critical situation that requires more intervention from mental health professionals to reduce symptoms in the community.

## Data Availability

All data generated or analyzed during this study are included in this published article.

## Abbreviations

OCD: Obsessive–compulsive disorder
COVID: Coronavirus disease
